# The Role of Prevolitional Processes in Video Game Playing: A Test of the Model of Goal-Directed Behavior and the Extended Model of Goal-Directed Behavior

**DOI:** 10.5964/ejop.v14i4.1565

**Published:** 2018-11-30

**Authors:** Bibiána Kováčová Holevová

**Affiliations:** a Department of Psychology, Pavol Jozef Šafárik University, Košice, Slovakia; Department of Psychology, Webster University Geneva, Geneva, Switzerland

**Keywords:** video games, prevolitional processes, the model of goal-directed behavior, the extended model of goal-directed behavior

## Abstract

The aim of the current study is to investigate the relationship between prevolitional processes and video game playing. Models of attitude, the model of goal-directed behavior (MGB) and the extended model of goal-directed behavior (EMGB) are tested with structural equation models to analyze the process that leads to video game playing. More specifically, the roles of affective, motivational, habitual processes in video game playing and the goal underlying video game playing are examined. The participants were 210 video game players who completed measures of Attitude, Subjective Norms, Perceived Behavioral Control, Behavioral Desire, Anticipated Emotions, Intention to Play, intensity of actual and past video game playing (Playing Behavior and Past Playing Behavior) and Goal Desire. The results showed that the initial MGB did not achieve a satisfactory fit and thus, a revised model with more acceptable fit was proposed. It was found that anticipated emotions and attitude are significant predictors of desire to play; desire to play, perceived behavioral control, subjective norms and attitude are significant predictors of intention to play and intention is a significant predictor of playing behavior (actual playing time). Moreover, past playing is a stronger significant predictor of behavior itself than of prevolitional processes in video game playing. Goal desire within the EMGB is a significant predictor of desire to play and the relationship of goal to playing behavior is indirect. Nevertheless, goal desire has an important role in the prevolitional processes of video game playing. In the discussion, potential explanations are further explored.

Video games have become increasingly popular over the last years and for many (especially young) people they are part of everyday life. The internet and technological innovations have contributed to the rapid development of video games. Video games have become more complex and sophisticated since their first introduction with more realistic graphics and storylines. Broadband internet has enabled gamers to play and interact online with each other. Due to the constant increase in video game playing and its popularity (e. g. Brand, Todhunter, & Jervis, 2017; Cummings & Vandewater, 2007; Hofferth, 2009; Rideout, Foehr, & Roberts, 2010; Ward, 2012), it is important to examine which variables play a role in the process of developing and maintaining game behavior. The aim of this study is therefore to research the prevolitional processes that lead to playing games. The study tests two theoretical models in the context of game behavior in order to identify which factors contribute to playing behavior. Perugini and Bagozzi (2001, 2004a) have proposed the model of goal–directed behavior (MGB) and the extended model of goal-directed behavior (EMGB) and confirmed that they have better predictive power than the previous theory of planned behavior (TPB, Ajzen, 1991). There have been four main areas of improvement: motivational processes, affective processes, automatic processes and means-end analyses. The study proposes to investigate their influence by applying the MGB and the EMGB to predict video game playing.

## Theory of Planned Behavior (TPB) and Video Game Playing

According to the TPB, the proximal cause of behavior is the intention to perform that given behavior. Besides intention, there are three fundamental constructs: (1) the attitude towards the behavior that corresponds to the degree to which a person has a favorable or unfavorable evaluation or appraisal of the behavior in question; (2) subjective norms (SN) which involve the perceived social pressure to perform or to not perform the behavior; and (3) perceived behavioral control (PBC), defined as the perceived ease or difficulty of performing the behavior. PBC is also posited as having a direct influence on behavior to the extent that it corresponds to actual behavioral control (Ajzen & Madden, 1986). According to the TPB, people act in accordance with their intentions and perceptions of control over their behavior, whereas intentions in turn are influenced by attitudes toward the behavior, SN and PBC. From an empirical point of view, the performance of the TPB has been assessed in a number of meta-analytic studies. Armitage and Conner (2001) examined 185 empirical tests of the TPB and found that the TPB accounted on average for 39% of the variance in intention and 27% of the variance in behavior.

However, little research has been devoted to the application of this model in the attitude towards video game playing. Haagsma, King, Pieterse, and Peters (2012) tested the utility of the theory of planned behavior model in video gaming activity and in explaining problematic video-game use among young Dutch people. The results showed that the TPB variables only explained 9% of the variance in intention. Attitude and PBC emerged as significant predictors whereas SN was not significant. PBC and intention had a significant influence on playing time and PBC and playing time had a significant influence on problematic video-game use. These authors also tested the TPB model using longitudinal data. They found that attitude, PBC and intention only explained 6% of the variance in playing time and 19% of the variance in problematic video-game use six months later (after controlling for playing time at the follow-up). Overall, PBC emerged as the most significant predictor of problematic video-game use over time. Despite the fact that the TPB model obtained statistical significance, the authors admitted that the total variance explained by this model was relatively low. Another study (Kováčová Holevová, 2017) showed that the TPB variables explained 31% of the variance in intention to play and 11% of the variance in playing time. Attitude and SN emerged as significant predictors of intention and intention was a significant predictor of playing time. Contrary to previous study, PBC was measured broader (not only perceived as easy or difficulty, but also as perceived control over behavior) and was not the significant predictor in either the intention to play or playing behavior. Indeed, the TPB does not take into account some prevolitional processes and is one of the reasons why Perugini and Bagozzi (2001, 2004a) proposed other models of attitude.

## The Model of Goal–Directed Behavior (MGB) and the Extended Model of Goal–Directed Behavior (EMGB)

Perugini and Bagozzi (2001, 2004a) expanded the TPB and suggested four main areas of improvement a) motivational processes, b) affective processes, c) automatic processes, and d) means-end analyses. They developed two models called the model of goal-directed behavior (MGB) and its extension, the extended model of goal-directed behavior (EMGB). They offer alternative views of the prevolitional decision-making processes.

### Motivational Processes

Perugini and Bagozzi (2001, 2004a) claimed that the first weak area of the TPB was the motivational processes in decision making. While attitudes, SN and PBC provide reasons for acting, they do not incorporate explicit motivational content needed to induce the intention to act. The authors proposed that desire is a stronger predictor of intentions than attitudes, SN and PBC. Desire is defined as the personal motivation or wish to perform an action. Based on their proposition, desire is a key construct introduced in the MGB. Desires perform energizing and transformative functions for the antecedents of decision making and represent the most proximal determinants of intentions. From this perspective, the usual TPB predictors do not directly determine behavioral intentions, but rather do so indirectly through desires.

The fact that one desires to perform a certain action does not necessarily imply that he/she will intend to do it. People often have desires, fleeting or otherwise, that they never intend to act upon. Intention and desire are distinct and Perugini and Bagozzi (2004b) proposed that desires are typically less doable, more abstract, less connected to actions and more future-oriented than intentions (see Leone, Perugini, & Ercolani, 2004; Perugini & Bagozzi, 2001, 2004a). Empirically, there is also correlational and experimental evidence supporting the distinction between desire and intention. In their meta-analysis of the TPB studies, Armitage and Conner (2001) showed that attitudes, SN and PBC significantly predicted more variance in desire than in intentions, and that intention was a better predictor of behavior than desire.

### Affective Processes

The original conception of the TPB mostly focused on the evaluative aspect without explicitly dealing with the specific contribution of the affective and cognitive components. Among the determinants of desire, the MGB also introduced the concept of anticipated emotions as predictors of intentions to act (Perugini & Bagozzi, 2001). Anticipated emotions represent a form of counterfactual (prefactual) thought processes where the emotional consequences of achievement; positive anticipated emotions (PAE) and failure; negative anticipated emotions (NAE) are appraised.

The authors also distinguish between attitude and anticipated emotions (Perugini & Bagozzi, 2004a). An attitude, either affective or cognitive, is typically constant over reasonable periods of time. By contrast, the processes behind the functioning of PAE and NAE are more dynamic and contingent on one’s appraisal of achievement or failure. This changes from time to time, depending on the context.

### Automatic Processes

The empirical evidence supports the importance of constructs such as intention and volition in predicting human behavior. On the other hand, there is evidence that automatic processes play an important role in human cognition and that they can direct behavior (Bargh, Chen, & Burrows, 1996; Bargh, Gollwitzer, Lee-Chai, Barndollar, & Troetschel, 2001; Chartrand & Bargh, 1999; Dijksterhuis & van Knippenberg, 1998).

Aarts and Dijksterhuis (2000) define habit as a form of goal-directed automatic behavior, which is activated automatically by the presence of relevant environmental cues, provided that the relevant goal is activated. Past behavior was introduced as a proxy for habit and automatic processes within the MGB and its effect on desire, intention and behavior is assumed (Perugini & Bagozzi, 2001). Based on the robust evidence of past behavior effects on intentions and behavior keeping the TPB variables constant suggests that good empirical reasons do exist for considering past behavior an independent predictor of intentions and behaviors (Conner & Armitage, 1998).

### Means-End Analyses

The last improvement of the TPB concerns goals. Goals play a central role in the explanation of many behaviors because these behaviors are chosen as a means of goal achievement (Gollwitzer & Moskowitz, 1996). In attitude theory, these behaviors have rarely been studied in relation to the goals for which they are performed. This has had practical meaning because the implicit assumption is that goals represent distal determinants of behavior whose influence is fully mediated by more proximal determinants of behaviors (Eagly & Chaiken, 1993).

Building on the MGB, Perugini and Bagozzi (2004a) proposed the extended model of goal-directed behavior (EMGB). They proposed that both goal desires (GD) and behavioral desires (BD) play a role in decision making concerning goal-directed actions. This follows from the assumption that most relevant behaviors can be better understood in light of the interplay between the goal and behavioral levels. It is maintained that the influence of a desire to achieve a certain goal will influence the desire to perform a certain behavior that is subjectively felt to be instrumental for goal attainment. As a result, BD will be the most proximal determinant of the intention to perform the behavior in question, and GD will have an indirect effect on intentions through behavioral desire. In sum, the only difference between the MGB and the EMGB is the inclusion of the concept of goal desire (GD) in the EMGB.

### Why Two Models?

According to Perugini and Bagozzi (2004a), the MGB and the EMGB are two models that can increase theoretical understanding. The MGB is the basic model and is used to predict and understand the behavior itself. It focuses on decision making processes and provides much more detail about motivational, affective and habitual processes in specific behavior. To the extent that one wants to emphasize both the behavioral and goal aspects of decision making, the EMGB is the most comprehensive model because it provides an additional ladder.

In the current study both these models (the MGB, the EMGB) are tested. Perugini and Bagozzi (2004a) have confirmed that the MGB has better predictive power than the TPB. Although the MGB has been tested for different behaviors such as weight control, studying or aggression (e.g. Leone et al., 2004; Perugini & Bagozzi, 2001; Perugini & Conner, 2000; Richetin, Richardson, & Boykin, 2011), there has been no research concerning the use of the MGB in explaining playing behavior. This is the reason to test the basic model for this new behavior first.

On the other hand, playing behavior can be better understood in light of the interplay between the goal and behavioral levels. The MGB can be enriched by focusing on the more distal determinants of an action such as the desire to achieve a given goal by means of some specific behavior and increase its predictive power. The EMGB incorporates the mechanisms and the processes through which the goal and the desire to achieve this goal influence behavior. Perugini and Bagozzi, (2004a) have confirmed that the introduction of goal desires is important as the construct always significantly predicts behavioral desires. However, the focus and the interest of this study is on both playing behavior and players ‘goal underlying this behavior. For this reason, the EMGB is also tested.

## The Present Study

In order to predict and understand playing behavior, this study is based on the (extended) model of goal – directed behavior that results from the well-known and widely adopted model of attitudes; the theory of planned behavior. In the MGB and the EMGB independent variables have been added as parallel predictors of behavioral intentions and behaviors (along with the established predictors in the TPB) and to which the TPB has not paid sufficient attention.

In testing this extended theoretical model (the MGB, the EMGB) in the context of gaming behavior, the factors which contribute to video game playing can be identified. A detailed analysis of this behavior can be made in the meaning of how strong various determinants are in the prediction of the prevolitional processes that lead to playing as well as in the prediction of playing behavior itself. In other words, what is the role of the established predictors of the TPB in players’ attitude to play, perceived social pressure to play or perceived behavioral control over playing. Moreover, do affective processes play a role and if so, whether a player´s positive or negative (anticipated) emotions play a more important role and whether playing habits or player´s goals underlying the behavior are significant determinants. Knowledge about the key factors that contribute to video game playing can help us understand what is behind this specific behavior and why playing video games is such a popular activity. Exploring the processes that develop and maintain playing behavior can also help to prevent the negative phenomenon related to increasingly intensive playing.

The present study has three main aims. The first aim is to test the MGB in video game playing. The predictive power of the MGB has been demonstrated in different behaviors from various domains (e. g. Perugini & Bagozzi, 2001; Perugini & Conner, 2000; Leone et al., 2004; Richetin et al., 2011). However, no findings concerning video game playing have been found. The MGB proposes that the proximal determinant of behavior is intention and the intention to perform instrumental behavior is primarily motivated by the desire to perform the act (BD). In turn, desires (BD) mediate the effects of attitudes, subjective norms (SN), perceived behavioral control (PBC) and anticipated emotions (PAE and NAE) on intentions.

The second aim is to investigate the role of past behavior in the MGB. Past behavior was introduced as a proxy for habit and automatic processes in the MGB (Perugini & Bagozzi, 2001). Yet, despite the evidence of past behavior effects on desires (Leone et al., 2004, Perugini & Bagozzi, 2001), intentions (Ouellette & Wood, 1998, Perugini & Bagozzi, 2001; Perugini & Conner, 2000) and behaviors (Leone et al., 2004; Ouellette & Wood, 1998; Perugini & Bagozzi, 2001), this construct has been the subject of much controversy (see Ajzen, 2002, 2004 for a different interpretation of past behavior effect). As such, some authors do not include past behavior when investigating prevolitional processes within the MGB that lead to certain behaviors and only focus on the deliberative processes (e. g. Richetin et al. (2011) did not include past behavior when investigating prevolitional processes that lead to aggressive behavior). However, the authors of the MGB and the EMGB have concluded that “…the evidence of the role played by past behavior in predicting future behavior is very strong. However, there is a range of opinions concerning its role in explaining future behavior. Either way, we believe that any comprehensive decision making model in this field should include past behavior, if for no other reason than to improve the prediction of intentions and behavior and/or control for unmeasured determinants” (Perugini & Bagozzi, 2004a, p. 174). This is the reason why a further aim of the current study is to investigate more thoroughly the role of past playing behavior in prevolitional processes that lead to actual video game playing as well as in the actual playing behavior itself.

Considering that goals may be an important part of playing behavior, the third aim was to investigate their influence by applying the EMGB (Perugini & Bagozzi, 2004a) in order to predict this behavior. The EMGB incorporates the mechanisms and processes through which the goal and the desire to achieve this goal influence behavior. More specifically, the EMGB suggests that the desire towards a distal goal (i.e., GD) such as the desire to have fun or to win over others elicits the desire towards a proximal goal (i.e., BD) such as the desire to play a video game. This, in turn, induces an intention to play video games that finally leads to playing behavior. The study intends to show that the goal one wants to achieve by video game playing does not directly relate to behavior but relates to behavior through the desire (BD) and intention to play video games.

Although the study is interested in applying the MGB and the EMGB to video games playing, it is not being suggested that the MGB or the EMGB provide a general model or theory of playing behavior. The MGB and the EMGB are general models that focus on decision making processes and can be applied to a variety of behaviors. The MGB and the EMGB provide much more detail about cognitive, emotional or habitual processes in the specific action.

The following hypotheses were proposed:

Hypothesis 1: Behavioral desire (BD) will be related to attitude, SN, PBC and PAE and NAE.
Hypothesis 2: Intention and PBC will be significantly related to playing behavior.
Hypothesis 3: Past playing behavior will be significantly related to BD, intention and playing behavior.
Hypothesis 4: In the EMGB, goal desire (GD) will not be a direct predictor of playing behavior.
Hypothesis 5: In the EMGB, goal desire (GD) will be a significant predictor of behavioral desire (BD).

## Method

### Sample and Data Collection

210 participants who were Slovak video game players (181 men, 29 women) from 14 to 35 years old (*M* = 20.1; *SD* = 5.7) completed measures of Goal Desire, Attitude, Subjective Norms, Perceived Behavioral Control, Behavioral Desire, Anticipated Emotions (PAE and NAE), Intention to Play and actual intensity of video games playing (Playing Behavior) and intensity of video games playing 6 months ago (Past Playing Behavior).

51% of participants were secondary school students, 18.6% were university students and 30.5% were employees. Almost 32% of them were Counter Strike players, 19.5% were World of Warcraft players, 10.5% were League of Legends players, 6.2% were EVE online players and 4.3% were Overwatch players.

The participants were obtained through occasional and snowball selection.

### Measures

With the exception of behavior and past behavior, all the responses were on 5-point scales with 5 indicating a higher score on the construct. The measures were adopted from other research investigating behavior within the MGB and the EMGB (Leone et al., 2004; Perugini & Bagozzi, 2001; Richetin et al., 2011).

Goal Desire (GD). In order to measure GD, participants were first asked, “What do you think would be the most likely reason why you would play video games?” After the participants had indicated their own reasons, they then responded to the following items by putting his/her own reason instead of letter Y. The desire towards his/her goal was measured with three items (“How strongly would you characterize your desire to reason Y?” “How likely is your desire to reason Y?” and “The intensity of your desire to reason Y can be described as?”). The reliability of this measure was very good (the Cronbach’s alpha was .854).

Attitude. Participants were presented with the stem “I think that for me video games playing is?” followed by nine bipolar scales (bad-good, negative-positive, unpleasant-pleasant, punishing-rewarding, unenjoyable-enjoyable, unsatisfying-satisfying, uncool-cool, useless-useful, harmful-harmless). This achieved very good reliability (the Cronbach’s alpha was .812).

Subjective Norms (SN). SN were assessed by three items (“People who are important to me think I should play video games,” “People who are important to me would approve of my video games playing,” and “People who are important to me would be very happy if I play video games”) (the Cronbach’s alpha was .829).

Perceived Behavioral Control (PBC). PBC was assessed with five items (“How much control do you have over video games playing?” “Whether I play video games or not is completely up to me,” “Succeeding in video games is easy for me,” “Succeeding in video games is difficult for me,” (scored as reversed) and “If I wanted to, it would be easy for me to play video games”). The reliability of this measure was not satisfactory (the Cronbach’s alpha was .467). The last item in the above list was not related to the others and was eliminated (the Cronbach’s alpha of the remaining items was .600). The reliability of the adjusted measure was at the edge of acceptability and therefore was not excluded from the measures tested in the models.

Anticipated Emotions. Positive Anticipated Emotions (PAE) were measured with five items. Participants indicated how delighted, proud, happy, pleased and satisfied they would feel if they succeeded in playing video games. Negative Anticipated Emotions (NAE) were also measured with five items. Participants indicated how disappointed, agitated, guilty, regretful and frustrated they would feel if they failed in playing video games. The reliabilities were high for both Negative (Cronbach’s Alpha was .933) and Positive Anticipated Emotions (the Cronbach’s alpha was .896).

Behavioral Desire (BD). BD was measured by three items (“How strongly would you characterize your desire to play video games” “I desire to play video games,” and “Video games playing is something that I desire to do”). Very good reliability was obtained (the Cronbach’s alpha was .855).

Intention. Intention was assessed by three items (“I will play video games,” “How likely is it that you will play video games?,” and “I intend to play video games”). The reliability of this measure was satisfactory (the Cronbach’s alpha was .789).

Playing Behavior and Past Playing Behavior. These were measured by the questions “Approximately how many hours over the week do you play video games these days?” (Playing Behavior) and “Approximately how many hours over the week did you play video games 6 months ago?” (Past Playing Behavior).

Participants also completed questions about the kind of video game that they played most frequently, their gender, age as well as choosing whether they were secondary school students, university students or employed.

## Results

### Preliminary Analyses

Age did not correlate with attitude, goal desire, subjective norms, negative anticipated emotions, behavior desire, intention to play, perceived behavioral control or playing behavior. Age significantly correlated only with positive anticipated emotions (*r* = -.146, *p* = .035) and past playing behavior (*r* = -.186, *p* = .007). It means the older video games players are, the fewer the hours they played video games in the past and the fewer the positive anticipated emotions they experience when they successfully play video games.

Female and male players did not differ in attitude, goal desire, subjective norms, negative anticipated emotions, positive anticipated emotions, behavioral desire, intention to play, perceived behavioral control or playing behavior. Female and male players only differed in past playing behavior (*t* = 2.980, *p* = .003). It means that the male players (*M* = 22.3 hours per week, *SD* = 13.8) reported a higher intensity of playing in the past than the female players (*M* = 14.8 hours per week, *SD* = 12.9). The descriptive characteristics measuring the variables are listed in Table 1.

**Table 1:** Means and Standard Deviations of the Components of the MGB and the EMGB (N = 210).

Component of the models	All players	
	*M*	*SD*
Intention to play	3.48	0.96
Behavioral desire to play	2.96	0.99
Attitude	3.91	0.59
Subjective norms	2.76	0.88
Perceived behavioral control	4.10	0.65
Positive anticipated emotions	3.52	0.96
Negative anticipated emotions	2.14	0.95
Goal desire	3.49	0.95
Playing behavior (hours per week)	18.00	12.68
Past playing behavior (hours per week)	21.20	13.93

Reasons for playing video games include the reasons (goals) participants listed for video games playing. The goals reported by the participants were: to have fun (31.4%), to avoid being bored (18.1%), to be with friends (15.2%), relax (10%), to escape from worry (10%), to win over other players (7.1%) and to advance in the game (6.2%).

### Tests of the MGB Applied to Video Games Playing

The models (the MGB and the EMGB further) were formally tested with structural equation models using AMOS 20. At first a full structural equation model was used in order to investigate the goodness of fit for the MGB. The predictive power of the model was tested by examining the amount of variance explained (R2) in the criteria (i.e., BD, intention, and behavior).

**Figure 1:**
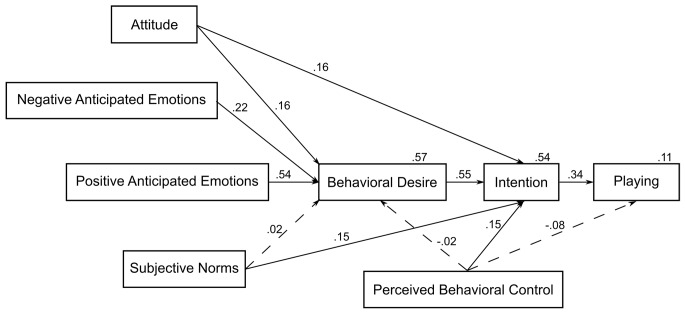
Parameters estimates for the MGB (without past behavior) applied to video game playing (*N* = 210).

Goodness of fit. Structural equation modeling was used to test the MGB. Attitude, subjective norms (SN), positive and negative anticipated emotions (PAE and NAE) and perceived behavioral control (PBC) were included as predictors of behavioral desire (BD). BD and PBC were included as predictors of intention. Intention and PBC were included as predictors of behavior (actual intensity of playing time). The initial model showed a poor fit (χ*^2^*(9, *N* = 210) = 33.045, *p* < .001, χ^2^*/df* = 3.672, CFI = .953, TLI = .852, NFI = .938, RMSEA = .113, PCLOSE = .006). Post hoc modification indices suggested an improved fit by direct effect from attitude and subjective norms to intention. The respecified model generated an adequate fit (χ^2^(7, *N* = 210) = 14.292, *p* = .046, χ^2^*/df* = 2.042, CFI = .986, TLI = .942, NFI = .973, RMSEA = .071, PCLOSE = .221). The standardized parameter estimates for the MGB are reported in Figure 1 (the correlations among predictors are omitted for the sake of simplicity). The results showed that the variables explained 57% of the variance in BD. Attitude, PAE and NAE emerged as significant predictors whereas SN and PBC were not significant. Therefore, Hypothesis 1 was partially supported. BD and PBC together with attitude and SN explained 54% of the variance in intention and intention explained 11% of the variance in playing behavior. PBC was not a significant predictor of playing behavior. Hypothesis 2 was also partially supported.

**Figure 2:**
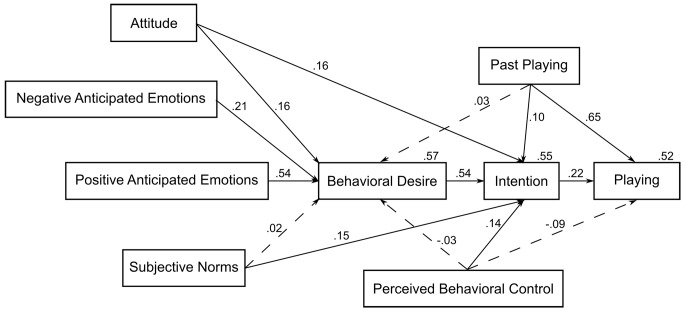
Parameters estimates for the MGB applied to video game playing (*N* = 210).

In the second step, past playing behavior as a predictor of behavioral desire, intention to play and playing behavior were added in order to examine the additional influence of this construct. Figure 2 presents a graphical representation of the results of the final model (the correlations among predictors are omitted for the sake of simplicity). Past playing was a significant predictor of intention and playing behavior and had a non-significant relationship with the desire to play (BD). The addition of past playing behavior slightly increased the amount of variance explained in intention (from 54% to 55%) but considerably (from 11% to 52%) in playing behavior (the obtained model fit was relative satisfactory χ^2^(7, *N* = 210) = 16.018, *p* = .025, χ^2^*/df* = 2.288, CFI = .986, TLI = .927, NFI = .976, RMSEA = .079, PCLOSE = .152). After entering past behavior, the beta weights form intention to behavior changed from .34 to .22. The statistical significance for any variables did not change. Hypothesis 3 was partially supported.

### Tests of the EMGB Applied to Video Game Playing

**Figure 3:**
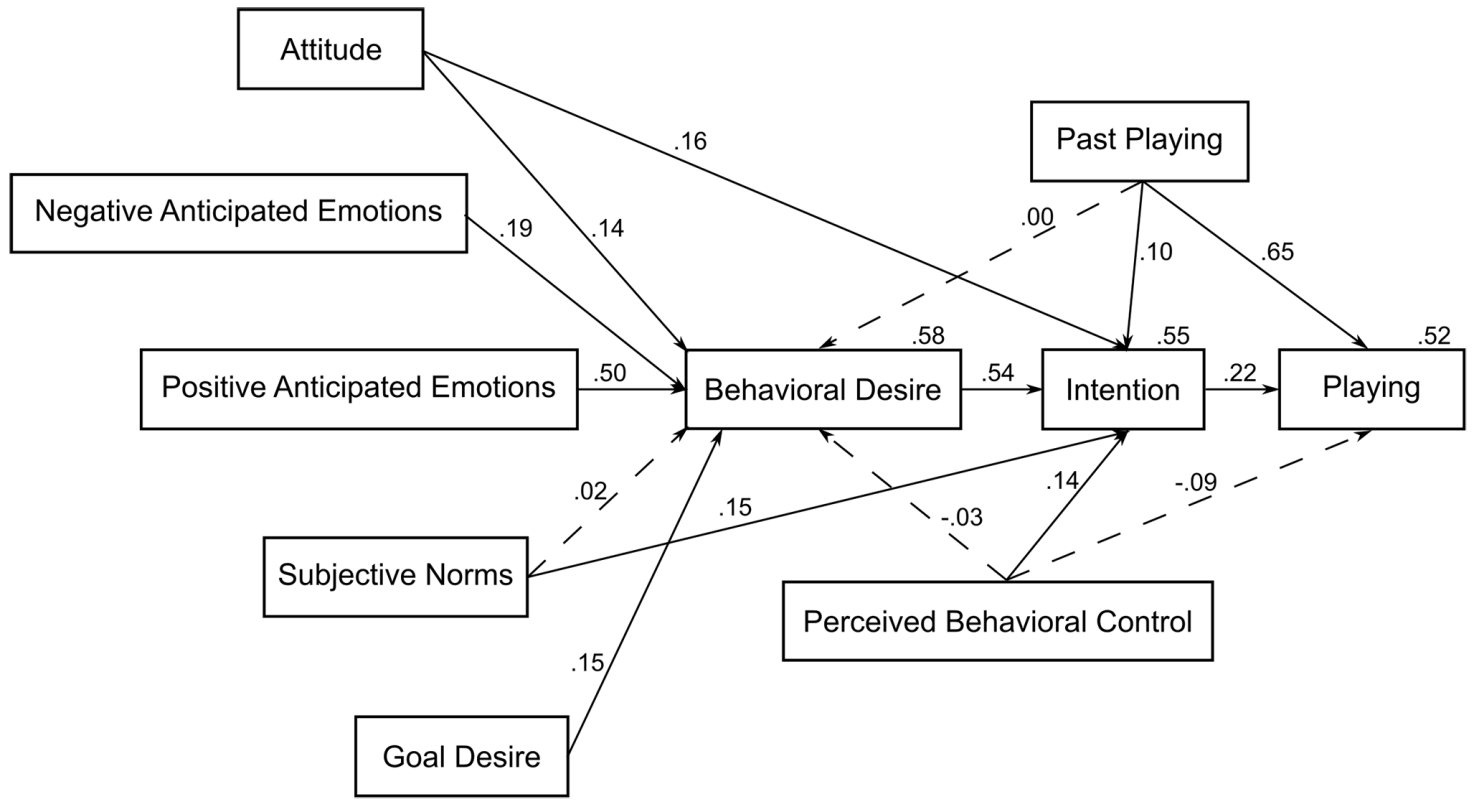
Parameters estimates for the EMGB applied to video game playing (*N* = 210).

The addition of goal desire (GD) as a predictor of behavioral desire (BD) slightly but significantly increased the amount of variance explained in BD (from 57% to 58%) (the model showed an adequate fit χ^2^(9, *N* = 210) = 17.552, *p* = .041, χ^2^*/df* = 1.95, CFI = .988, TLI = .940, NFI = .977, RMSEA = .067, PCLOSE = .236). After entering GD, the beta weights for attitude, PAE and NAE changed a little and became lower. However, the statistical significance for none of the variables changed. Goal desire related to playing behavior indirectly through behavioral desire. The standardized parameter estimates for the EMGB are reported in Figure 3 (the correlations among predictors are omitted for the sake of simplicity). Hypotheses 4 and 5 were supported.

## Discussion

This study was designed to examine the prevolitional processes of video games playing (especially actual intensity of this behavior measured by actual playing time) and to investigate the goals underlying the behavior as well as the role of those goals in the determination of the desire to play (BD) and playing behavior (actual intensity of video game playing time). With structural equation modeling, the goodness of fit of the MGB and the EMGB model were tested.

The first aim of this study was to investigate the motivational and affective processes within the MGB that lead to video game playing. It was found that anticipated emotions (PAE, NAE) and attitude were significant predictors of behavioral desire (BD); BD and perceived behavioral control (PBC) were significant predictors of intention and intention was a significant predictor of behavior. The desire to play (BD) did not fully mediate the direct effect of subjective norms (SN), PBC and attitude on the intention to play. However, the fit of the initial model was not satisfactory (χ^2^(9, *N* = 210) = 33.045, *p* < .001, χ^2^*/df* = 3.672, CFI = .953, TLI = .852, NFI = .938, RMSEA = .113, PCLOSE = .006). Adding direct paths from attitude and SN to intention improved the fit of the model (χ^2^(7, *N* = 210) = 14.292, *p* = .046, χ^2^*/df* = 2.042, CFI = .986, TLI = .942, NFI = .973, RMSEA = .071, PCLOSE = .221).

The MGB (Perugini & Bagozzi, 2001) proposes that desire mediates the effects of other predictors on intention. The full mediation hypothesis was confirmed by Perugini and Bagozzi as well as gathering support in the current data too. It was found that BD mediated the effect of PAE and NAE on intention and did not fully mediate the effect of attitude on intention. SN and PBC were not significant predictors of desire for playing video games (BD). Nevertheless, even when the aforementioned direct effects from attitude and SN to intention were included, BD was still by far the strongest predictor.

Consistent with previous findings (Leone et al., 2004; Perugini & Bagozzi, 2001, 2004a; Richetin et al., 2011), the significant predictors of BD were PAE and NAE. The anticipation of the positive feelings one would have, if one succeeded in video game playing and the anticipation of the negative feelings one would have, if one failed in video game playing are associated with the desire to play video games (BD). The greater variance explained by PAE compared to NAE in BD echoes the main reported goals underlying video game playing. Indeed, the anticipation of how delighted, proud, happy, pleased or satisfied a player would feel if he or she succeeded in video game playing corresponds to the improved affect one aims to achieve (e.g. to have fun, to win over the other players or to be with friends...) by engaging in video game playing. The anticipation of how disappointed agitated, guilty, regretful or frustrated a player would feel if he or she failed in video game playing also contributed to the BD.

Although Perugini and Bagozzi (2001) assume in the MGB that the effect of attitude on intention is mediated by BD, in some cases and for some behaviors, the mediation by BD may not be complete (e.g. Leone et al., 2004). Given that previous research has demonstrated that attitude influences intention, it is theoretically meaningful to allow a direct path from attitude to intention. It was found that the attitude to play video games was a significant predictor of BD, although attitude also had a direct effect on the intention to play video games.

In contrast to the full mediation role of BD, SN was considered by Bagozzi (1992) as capable of directly energizing intention. Leone et al. (2004) investigated studying behavior (studying handbooks as instrumental behavior leading to goal attainment – passing an SPSS Windows mastery test) and found support for Bagozzi’s hypothesis. The current results also support the direct effect of SN on intention. This might represent the motivation to behave in accordance with a participant’s role and to confirm the participant’s self-concepts (Carver, 1996). It is likely that video game playing is a defining characteristic of the role identities of the participants.

This study also sheds light on the role of PBC. PBC was significantly related to intentions although PBC has no significant connection to BD or to behavior. The participants were players and obviously considered video games playing as a rather easy behavior (*M* = 4.1, *SD* = 0.65). Under these circumstances, self-efficacy beliefs can play a major role in influencing intention. As Perugini and Conner (2000) have pointed out, self-efficacy processes can have an impact by themselves on forming intentions, but this self-efficacy belief may be partly independent from the desire to engage in that behavior. High self-efficacy perceivers may consider rewarding the anticipated feeling of mastery that they expect to experience by performing the behavior successfully, and may therefore build their intentions on their own self-efficacy perceptions. It is likely for a player to intend to enact the video game playing when he or she believes himself or herself capable of mastering.

Leone et al. (2004) also investigated practicing behavior (practicing with a package as instrumental behavior leading to goal attainment – passing an SPSS Windows mastery test) and also found a direct connection between PBC and intention. Based on these results (the importance of perceptions of control and self-efficacy in forming intention) the authors recommended that when the behavior to be performed implies direct contact or practice with instruments or devices, the perceived ease (or difficulty) of the behavior may be manipulated to facilitate effective learning and performance. Playing video game is a type of behavior at which practice with instruments or devices is needed. It may be a reason why players´ perceived control over his or her playing plays a significant role in forming the intention to play. On the other hand, differences in the pattern of predictors may be found in different studies and across different behaviors (Leone et al., 2004; Perugini & Bagozzi, 2001; Richetin, Perugini, Adjali, & Hurling, 2008; Richetin et al., 2011). However, this leaves the question of which should be significant in different domains as an empirical matter. The differences in the importance of predictors might be due to a different context of behavior (for example playing video game belongs to leisure activities versus other more onerous tasks) but more research is needed to verify this speculation.

In sum, under some circumstances, the mediational power of BD might not mediate all the effects of the MGB constructs on intention. While what is desired is often intended, intention still might be based directly on reasons and beliefs concerning the behavior. Video game playing might be intended because players are believed to be normatively appropriate and therefore it is crucial for one’s role identity. The development of an intention to play can be facilitated if the player feels capable of enacting the behavior. In such cases, the motivational input to intention comes from SN and PBC and these motivational inputs can influence intention directly without being completely mediated by BD.

The second aim of the study was to investigate the role of past behavior in the MGB that leads to video game playing. Past behavior was introduced as a proxy for habit and automatic processes in the MGB (Perugini & Bagozzi, 2001, 2004a) and may play a significant role in prevolitional processes that lead to video game playing as well as in the playing behavior itself.

It was found that past playing behavior had a significant relationship with intention and playing behavior but did not have significant relationship with BD. The obtained model fit was relative satisfactory (χ^2^(7, *N* = 210) = 16.018, *p* = .025, χ^2^*/df* = 2.288, CFI = .986, TLI = .927, NFI = .976, RMSEA = .079, PCLOSE = .152). Past playing has a much stronger effect on playing behavior itself than on prevolitional processes that lead to playing behavior and increased the amount of variance explained especially in behavior. It seems meaningful because automatic processes that are behind (habitual) past playing can directly influence actual video games playing without being mediated by the desire to play (BD) and being mediated by intention only marginally. Habit strength and its direct effect on media use is also emphasized in another model; the model of media attendance (LaRose & Eastin, 2004). Although Ajzen (1991) argued that the inclusion of PBC should preclude the need for past behavior, in that PBC should mediate any residual effects of past behavior, the current research has found that past playing still predicts the intention to play and especially actual playing behavior in tests of the MGB. It is likely that habitual processes have a significant role in playing behavior and should not be omitted.

Considering that goals may be an important part of video game playing, it was proposed that their influence should be investigated more thoroughly by applying the EMGB proposed by Perugini and Bagozzi (2004a) to predict the actual intensity of video games playing. The third aim of this research was to use the EMGB to determine whether the goal one wants to achieve when playing video games had an indirect rather than a direct relationship with behavior through BD and intention. The obtained model fit was satisfactory (χ^2^(9, *N* = 210) = 17.552, *p* = .041, χ^2^*/df* = 1.95, CFI = .988, TLI = .940, NFI = .977, RMSEA = .067, PCLOSE = .236). The current results confirmed that video game playing can be motivated by many different goals. The contribution of GD on BD was significant and the desire towards the goal one wants to achieve by video games playing (GD) did not relate directly to the emergence of the behavior. However, it had an indirect association through the desire towards video game playing (BD). In other words, wanting to have fun or win over other players will not directly increase the likelihood of actual video game playing, but rather relates to the desire to play video games (BD). The results also reveal that the desire to have fun or win over other players (GD) not only predicts the desire to play video games (BD), but has a more important role than more intuitively compelling and well-established constructs. Indeed, SN and PBC did not play a critical role, whereas PAE, NAE and attitude were significant predictors of one’s desire to play video games (BD). In fact, the emotional and motivational processes that were taken into account by including anticipated emotions and GD, respectively, together with attitude, were three of the main significant predictors of BD. One should also note that there is a consistency between the goals expressed by participants in this study and the significant contribution especially of PAE in determining the desire to play video games.

### Limitations and Future Research

The limitations of this study need to be acknowledged. This study is correlational and therefore may shed light only indirectly on the causal mechanisms underlying decision-making processes. Nevertheless, the results were consistent with the hypothesized theoretical framework. Future experimental studies could manipulate the key variables explicitly. It also needs to be acknowledged that procedures used to measure some constructs may be improved. Past behavior was introduced as a proxy for habit and automatic processes in the MGB (Perugini & Bagozzi, 2001, 2004a) although this construct has been the subject of much controversy (Ajzen, 2002, 2004). Maybe other means of detection of habitual or automatic processes within the MGB or the EMGB should be investigated. PBC was another construct where the procedure used to measure it may be improved. Although similar problems with reliability have been found in other studies (Richetin et al., 2011), items measuring this construct are used in a lot of research investigating behavior within the MGB and the EMGB (Leone et al., 2004; Perugini & Bagozzi, 2001, 2004a; Richetin et al., 2011). However, past behavior had a stronger effect on playing behavior and PBC had a stronger effect on intention to play. The limitation of this study is also the relatively small number of female respondents compared to male respondents. Although the disproportion in the sample is likely to reflect the real disparity between male and female players and non-significant differences between them were confirmed in most of the variables, further research with a higher prevalence of female players is needed to confirm the current findings.

### Conclusion

Due to the increase in gaming behavior, it is important to research the prevolitional processes that lead to playing games. The study tested two theoretical models: the model of goal–directed behavior (MGB) and the extended model of goal-directed behavior (EMGB) (Perugini & Bagozzi, 2001, 2004a) in the context of playing behavior in order to identify which factors contribute to video game playing. Desires, goal-anticipated emotions, and past behavior broaden the representation of goal-directed behaviors provided by the theory of planned behavior (TPB, Ajzen, 1991) and increase the prediction of intention and behavior. The MGB and the EMGB have been tested in different behaviors but no findings concerning playing behavior have been found. Therefore, the second benefit of this study represents the assessment of these models in the context of a new behavior video game playing.

The comparison between the predictive power of the tested models for (behavioral) desire to play, intention to play and playing behavior have shown that the extended models (the MGB with past playing behavior and the EMGB) accounted for more variance than the basic MGB. When applied to video game playing, the basic MGB without past playing behavior (the model of attitude that considers behavior as planned and largely determined by the intention to perform it) explained 57% of the variance for the desire to play, 54% of the variance for intention to play, but only 11% of the variance for playing behavior. The MGB with past playing behavior accounted for more explained variance especially in playing behavior and the EMGB accounted for slightly more variance in desire to play.

Practitioners may benefit from knowing the processes that lead to playing games. The determinants of the MGB or the EMGB have a different role in this process. The results have suggested that anticipated emotions and attitude seem to be connected with more distal determinants of playing behavior; the desire to play. This construct does not necessarily imply that the player will intend to play. However, the desire to play was the strongest predictor of a more proximal determinant of playing behavior and together with perceived control/perception of control over playing (PBC), perceived social pressure to play (SN) and attitude, had a direct effect on the intention to play. Only two determinants had a direct effect on actual playing behavior; the intention to play and past playing behavior. Therefore, it is necessary to know a player´s playing habits when analyzing the process of developing and maintaining playing behavior. Moreover, past playing was a stronger predictor of playing behavior itself than of prevolitional processes that lead to playing games. The results have also suggested that the player´s goals played an important role in the prevolitional processes of video game playing. While their relationship to playing behavior was indirect, goal desire had an important role in the desire to play.

Based on the findings, there is an assumption that habitual or automatic processes in the prevolitional processes that lead to playing should not be omitted because they can play a role in playing behavior. The current results also support the view that the interplay between goal and behavioral levels of analysis represents an important element in understanding the decision making process that leads to video game playing.
